# Star Polymers as a Reducing Agent of Silver Salt and a Carrier for Silver Nanoparticles

**DOI:** 10.3390/ma18133009

**Published:** 2025-06-25

**Authors:** Katarzyna Szcześniak, Grzegorz Przesławski, Jakub Kotecki, Weronika Andrzejewska, Katarzyna Fiedorowicz, Marta Woźniak-Budych, Maciej Jarzębski, Piotr Gajewski, Agnieszka Marcinkowska

**Affiliations:** 1 Faculty of Chemical Technology, Poznan University of Technology, Berdychowo 4, 60-965 Poznan, Poland; grzegorz.przeslawski@doctorate.put.poznan.pl (G.P.); jakub.kotecki@student.put.poznan.pl (J.K.); piotr.gajewski@put.poznan.pl (P.G.); agnieszka.marcinkowska@put.poznan.pl (A.M.); 2 NanoBioMedical Centre, Adam Mickiewicz University in Poznan, Wszechnicy Piastowskiej 3, 61-614 Poznan, Poland; weronika.andrzejewska@amu.edu.pl (W.A.); katfie1@amu.edu.pl (K.F.); marta.budych@amu.edu.pl (M.W.-B.); 3 Department of Physics and Biophysics, Faculty of Food Science and Nutrition, Poznan University of Life Sciences, Wojska Polskiego 38/42, 60-637 Poznan, Poland

**Keywords:** star polymers, silver nanoparticles, bone cements, antibacterial activity

## Abstract

Star polymers—macromolecules featuring multiple arms radiating from a central core—offer unique potential for biomedical applications due to their tunable architecture, multifunctionality and ability to incorporate stimuli-responsive and biocompatible components. In this study, functional star polymers with oligo (ethylene glycol) methyl ether methacrylate (OEOMA) arms and 2-(dimethylamino)ethyl methacrylate (DMAEMA) core units were synthesized via atom transfer radical polymerization (ATRP) using the “arm-first” strategy. The star polymers were used as nanoreactors for the in situ reduction of silver nitrate to form silver nanoparticles (AgNPs) without additional reducing agents. UV–Vis spectroscopy confirmed the formation of spherical AgNPs with absorption maxima around 430 nm, and transmission electron microscopy revealed uniform particle morphology. These hybrid nanomaterials (STR-AgNPs) were incorporated into polymethyl methacrylate (PMMA)-based bone cement to impart antibacterial properties. Mechanical testing showed that the compressive strength remained within acceptable limits, while antibacterial assays against *E. coli* demonstrated a significant inhibition of bacterial growth. These findings suggest that STR-AgNPs serve as promising candidates for infection-resistant bone implants, providing localized antibacterial effects while maintaining mechanical integrity and biocompatibility.

## 1. Introduction

Star polymers are macromolecules characterized by a branched architecture. In such molecules, three or more polymeric arms are covalently attached to a central core. The central core may consist of an atom, a small molecule, a nanoparticle, a nanogel, etc. [1,2,3,4]. These polymers are classified into two types: regular star polymers with symmetric arms, and miktoarm star polymers with asymmetric architectures [5,6]. Various controlled polymerization techniques are used in the synthesis of star polymers, ranging from anionic [7] to radical polymerizations such as reversible addition−fragmentation chain transfer (RAFT) polymerization [8] and atom transfer radical polymerization (ATRP) [9,10]. These techniques are based on three strategies: “core-first”, “coupling-onto” and “arm-first” [9,11,12,13].

In the “arm-first” strategy, well-defined linear arms are first synthesized and then covalently bonded to a crosslinking agent to form the core. This allows for the precise control of arm lengths in the synthesis of star polymers with multiple arms and functionalities. However, the obtained star polymers typically exhibit statistical variation in the number of arms and a relatively broad molecular weight distribution (MWD, *M*_w_/*M*_n_ > 1.5). Additionally, the final product is often contaminated with residual unreacted linear polymer precursors. Consequently, it is important to purify the product through laborious fractionation techniques to obtain a star polymer with higher purity and a narrower MWD [14].

For biomedical applications, star polymers should exhibit a well-defined structure, characterized by a specific composition and a molecular architecture with emphasis on molecular weight (*M*_w_), MWD and number of arms [5,9]. They should also possess specific functionalities, responsiveness to stimuli, biocompatibility and either biostability or biodegradability. Nonionic and biocompatible poly (ethylene oxide) (PEO) macromolecules offer unique properties such as chemical stability, water solubility, non-toxicity and evasion of immune system recognition. Therefore, integrating PEO chains into star polymer architectures may provide additional benefits, including the presence of multiple functional groups, compared to linear PEO counterparts, thereby enhancing their suitability for these applications [15,16].

The unique structure of star polymers and the large number of active groups, as well as their high density, allows for easy modification to impart desired properties [17,18,19], such as cell adhesion [20,21,22] or antibacterial activity [23,24,25]. Vigliotta et al. [26] synthesized heterojunction star polymers with 2-(dimethylamino)ethyl methacrylate (DMAEMA) as antibacterial functional units. This made it possible to obtain antibacterial materials without diffusion, and the effective performance did not decrease even after 20 uses. Functional monomers like DMAEMA can also be incorporated into star polymers to form complexes with drugs or nucleic acid [27], or used as a reducing agent for silver ions in obtaining silver nanoparticles (AgNPs). This process can occur through the absorption of the Ag^+^ ions by tertiary amine groups via coordination interaction, followed by the in situ generation of AgNPs, without an additional reducing agent.

Lin et al. [28] demonstrated the synthesis of AgNPs via the in situ reduction of silver nitrate (AgNO_3_) in star-shaped polymeric micelles composed of poly (ε-caprolactone), DMAEMA and oligo (ethylene glycol)monomethyl ether methacrylate (OEOMA). The resulting AgNPs exhibited a spherical morphology with diameters ranging from 10 to 20 nm, regardless of the copolymer architecture or the AgNO_3_ concentration. In another study, Huang et al. [29] synthesized AgNP-decorated copolymer micelles with various copolymer topologies. Linear or four-arm star triblock copolymers composed of DMAEMA, 2-hydroxyethyl methacrylate (HEMA) and poly (ethylene glycol)methyl ether methacrylate (PEGMA) with comparable molecular weights and degrees of polymerization were prepared to reduce silver ions in situ. Computational modeling and experimental results showed that both types of micelles could produce AgNPs with monodisperse and spherical morphology. However, star copolymer micelles resulted in AgNPs with smaller average size, improved stability and higher antibacterial activity compared to linear structures, likely due to the greater stability of star copolymer micelles.

The ability to generate silver nanoparticles on a carrier opens up new possibilities for the application of AgNPs, e.g., in orthopedics. Nowadays, many people suffer from complicated bone fractures resulting from accidents or various diseases, such as bone cancer, bone degeneration and osteoporosis. In 2024, the National Institutes of Health (NIH) estimated that 3970 new cases of primary malignant bone cancers would be diagnosed in the United States, with about 2050 deaths. Moreover, as of 2020, osteoarthritis (OA) affected approximately 595 million people globally, representing about 7.6% of the world’s population [30], and a comprehensive systematic review and meta-analysis estimated the global prevalence of osteoporosis at 18.3% [31]. Most of these defects can be stabilized and treated with bone cements, but they are associated with a risk of infection and therefore require the use of antibacterial agents [32,33,34]. Silver nanoparticles are widely studied as antibacterial agents in bone cements [35,36,37,38]. As Wekwejt et al. [37] showed in their studies, the addition of silver nanoparticles imparts antibacterial efficacy; however, it worsens cytotoxicity. These studies indicate great potential in the antibacterial activity of AgNPs in bone cements; however, free AgNPs may be toxic for cells. This led us to try to incorporate STR-AgNPs into acrylate bone cement.

In this work, we synthesized functional star polymers with OEOMA arms and DMAEMA units in the core as potential reducing agents for the in situ generation of AgNPs for antibacterial applications. The star polymers were obtained using the ATRP technique and the “arm-first” method with DMAEMA content. Molecular weight, molecular weight distribution and size were analyzed. The reduction process was fully monitored by investigating the maximum absorption wavelength using UV–Vis spectroscopy. Additionally, the stability, morphology and particle size of the star polymers with silver nanoparticles (STR-AgNPs) were examined. The obtained STR-AgNPs were introduced to the bone cement, and the mechanical properties and antibacterial activity of the prepared materials were tested.

## 2. Materials and Methods

### 2.1. Materials

Oligo (ethylene glycol) methyl ether methacrylate (OEOMA_500_), 2-(dimethylamino)ethyl methacrylate (DMAEMA, 97%), ethylene glycol dimethacrylate (EGDMA, 98%), ethyl 2-bromoisobutyrate (EBiB, 99%), 1,1,4,7,10,10-hexamethyltriethylenetetramine (HMTETA, 97%), copper (II) bromide (CuBr_2_, 99%), copper (I) bromide (CuBr, 99.999% trace metal basis), silver (I) nitrate (AgNO_3_, >99%, Sigma Aldrich), ethanol (EtOH, 99.9%, p.a.) and methanol (MeOH, 99.9%, p.a.) were obtained from Sigma Aldrich, (St. Louis, MO, USA). The commercially available bone cement was Refabocin^®^ Plus Bone Cement (Zimmer Biomed, Warsaw, IN, USA). Acetone (Ace, 99.9%, p.a.) was from Avantor Performance Materials Poland (Gliwice, Poland).

### 2.2. Synthesis

**Figure 1:**
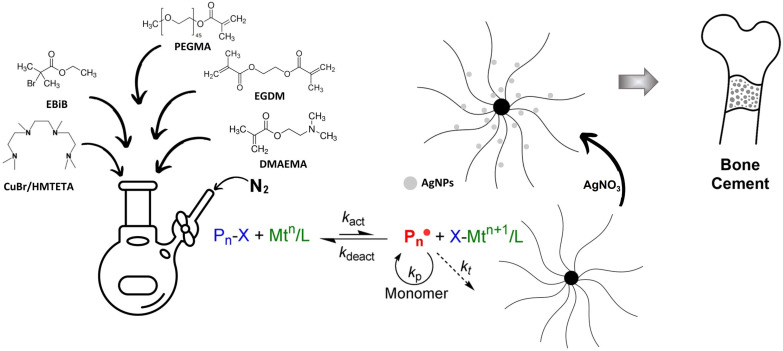
Schematic idea of the synthesis steps.

The synthesis scheme is presented in Figure 1, which shows the detailed production path of bone cement modified with star polymers on which silver nanoparticles are deposited. The silver nanoparticles are formed in situ through the reduction of silver ions complexed with the polymer matrix, resulting in a multifunctional biomaterial with enhanced antibacterial potential.

#### 2.2.1. Star Polymer Synthesis

The monomers (OEOMA_500_, DMAEMA, EGDMA) were passed through the basic alumina to remove the inhibitor. All other chemicals (EBiB, HMTETA and solvents) were used as received without further purification. The reagents were used in the molar ratio of [OEOMA_500_]_0_/[DMAEMA]_0_/[EGDMA]_0_/[EBiB]_0_/[CuBr]_0_/[CuBr_2_]_0_/[HMTETA]_0_ = 1/x/1/0.2/0.19/0.01/0.2, where x is 2 (STR_1) or 4 (STR_2). OEOMA_500_ (Mn = 526, 0.526 g, 1.0 mmol), DMAEMA (337 μL, 2.0 mmol or 674 μL, 4.0 mmol), EGDMA (189 μL, 1.0 mmol), HMTETA (54 μL, 0.2 mmol), CuBr_2_ (2 mg, 0.01 mmol), methanol (10 mL) and toluene (0.5 mL) were charged to a Schlenk flask. The reaction mixture was purged with N_2_ and then CuBr (27 mg, 0.19 mmol) was added to the flask under N_2_. The flask was sealed and immersed in a 60 °C oil bath. The deoxygenated initiator EBiB (29.6 μL, 0.2 mmol) was injected into the reaction mixture, via a nitrogen-purged syringe, through the side arm of the Schlenk flask under N_2_ [39]. At timed intervals, samples were withdrawn via a syringe fitted with stainless-steel needle to follow conversions of DMAEMA and EGDMA by gas chromatography (GC) analysis. The reaction was stopped after 24 h by exposure to air. The final products were purified with a dialysis membrane bag (MWCO 25 kDa) against methanol for 3 days.

#### 2.2.2. Silver Nanoparticles Synthesis

The obtained star polymers were dissolved at a concentration of 4 mg/mL in ethanol (EtOH), and two different mixtures of solvents were prepared in a weight ratio of 4:1, i.e., water/acetone (H_2_O-Ace) and ethanol/acetone (EtOH-Ace). Then, a solutions of star polymers in an appropriate solvent were mixed with a 0.35 M silver (I) nitrate solution prepared in the same solvent. The DMAEMA/Ag^+^ molar ratio was fixed at 1:1. Flasks were kept in the dark. The progress of reduction was monitored by UV–Vis spectroscopy, until silver began to precipitate on the walls of the vial in the form of a mirror, which meant that the star no longer complexed the silver nanoparticles that were formed. STR-AgNPs obtained in EtOH and EtOH-Ace were dried under vacuum, and STR-AgNPs obtained in H_2_O-Ace were frozen at −80 °C for 24 h and then freeze-dried.

#### 2.2.3. Synthesis and Mechanical Testing of Bone Cements

The PMMA bone cement Refabocin Plus was used as the base material. The STR-AgNPs were added in the amount of 2.5 wt.% to the powder phase. The powder phase and the liquid phase were manually mixed according to manufacturer’s specifications. After mixing, the bone cement dough was placed in cylindrical (12 mm × 6 mm) Teflon^®^ molds. Polymerization was carried out for 1 h at constant temperature (36.6 °C) and humidity (30%). The collected bone cement samples were removed from the mold and after 24 ± 2 h were subjected to a compressive strength test on a Zwick/Roell Z020 universal testing machine (Zwick AG, Ulm, Germany). A constant crosshead speed of 22.5 mm/min was used. The measurement was carried out until the specimen broke and was repeated at least for five samples. The results obtained for compressive strength and Young’s modulus were recalculated using a computer program dedicated to the Zwick/Roell Z020 testing machine.

### 2.3. Methods

#### 2.3.1. Gel Permeation Chromatography (GPC)

The molecular weight (*M*_n_) and dispersity (*Ð*) were measured relative to poly (methyl methacrylate) (PMMA) standards by gel permeation chromatography (GPC), conducted with an Agilent gel permeation chromatograph (GPC) (Agilent, Santa Clara, CA, USA) with THF as an eluent. The GPC was equipped with an RI, a multi-angle laser light-scattering (MALS) detector and PSS columns (Styrogel 105, 103, 102 Å); the process was conducted at 35 °C with a flow rate of 1 mL/min.

#### 2.3.2. UV–Vis Spectroscopy

UV–Vis spectroscopy measurements were carried out on a Jasco V-530 spectrophotometer (Jasco, Tokyo, Japan) using quartz cuvettes with an optical path length of 0.1 cm, at a temperature of 25 °C, in the wavelength range of 300–600 nm, at set time intervals.

#### 2.3.3. Transmission Electron Microscopy (TEM)

Transmission electron microscopy measurements were performed using a JEM-1400 microscope (JEOL, Tokyo, Japan) with an accelerating voltage of 120 kV. Aqueous solutions of polymers were prepared from powders and diluted to 1:100. Samples were spotted onto a commercially available Cu Lacey grid, without formvar, and air-dried at room temperature.

#### 2.3.4. Antibacterial Studies

The antibacterial activity of bone cements was evaluated against *Escherichia coli* (*E. coli*—ATCC 35281). The *E. coli* was incubated overnight in a lysogeny broth (LB) medium at T = 37 °C in the incubator with orbital shaking (230 rpm). Then, 50 µL of *E. coli* suspension was pipetted into tubes containing 5 mL of fresh LB and tablets: BC_STR_1-AgNPs and BC_STR_2-AgNPs. For the negative control samples, control (−A) was cultivated without antibiotics, while for the positive Rifampicin solution (1 mg/mL), control (+A) was added. Both strains were cultivated as before (in the incubator with orbital shaking at 230 rpm at T = 37 °C). At indicated time points, 100 µL of each sample suspension was transferred into 96-well plates, and OD measurement (in triplicates) was performed at λ = 600 nm using a plate reader (Anthos Zenyth 340rt, Anthos Labtec Instruments GmbH, Salzburg, Austria).

## 3. Results

Star polymers were prepared by ATRP using the “arm-first” method and macromonomer approach [39] with OEOMA_500_ as arms, EGDM as a crosslinker and DMAEMA as a functional monomer, to complex and reduce Ag^+^ ions. DMAEMA was used in two different amounts. STR-AgNPs were obtained in three various solvents at room temperature without exposure to the light. The process lasted a few days and was strictly monitored via UV–Vis spectroscopy. STR-AgNPs were characterized by UV–Vis spectroscopy and transmission electron microscopy (TEM).

### 3.1. Synthesis and Characterization of Star Polymers

**Figure 2:**
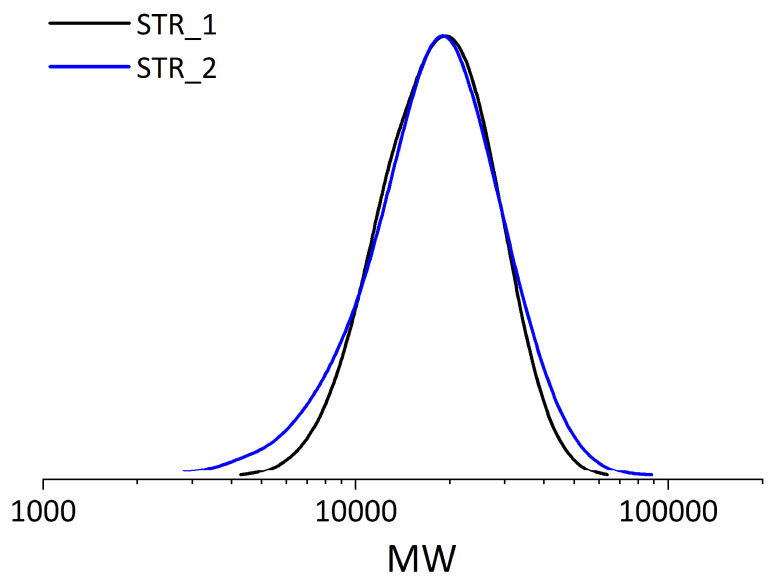
Star polymer size as a molecular weight by GPC.

The number-average molecular weight (*M*_n_) and dispersity (*Ð*) of the obtained star polymers were characterized by gel permeation chromatography (GPC) with a refractive index. STR_1 and STR_2 had *M*_n_1_ = 16,100 g/mol and *M*_n_2_ = 15,200 g/mol, respectively. The *Ð* values were 1.42 and 1.57, respectively. The absolute number-average molecular weights (*M*_n,abs_) obtained by multi-angle laser light-scattering (MALS) detector were 23,900 and 28,400, respectively. The observed difference in *M*_n_ is due to conventional GPC calibration based on the hydrodynamic volume of linear polymer standards. An accurate *M*_n_ for polymers with complex architectures, such as star polymers, can be obtained using MALS detectors [39,40]. The number of arms in STR_1 and STR_2 was calculated by comparing the *M*_n_ of the star polymer and the arm precursor based on the study of Cho et al. [39]; they were found to be 47 and 56, respectively. The results are shown in Table 1 and Figure 2. The NMR data are presented in Supplementary Material (Figure S1).

### 3.2. Studies of Silver Salt Reduction on Synthesized Star Polymers

**Figure 3:**
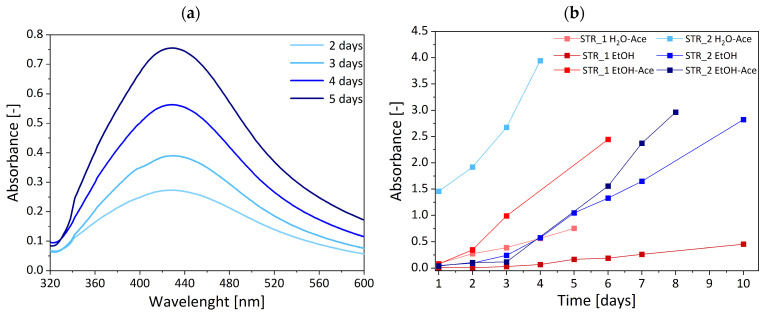
(**a**) UV–Vis spectra obtained during the reduction process of silver ions in H_2_O-Ace solution of STR_1, (**b**) dependence of the maximum absorbance of the characteristic absorption peak of silver nanoparticles on time for different solutions of STR_1 and STR_2. UV spectra were measured in quartz cuvette d = 0.1 cm at 25 °C.

**Figure 4:**
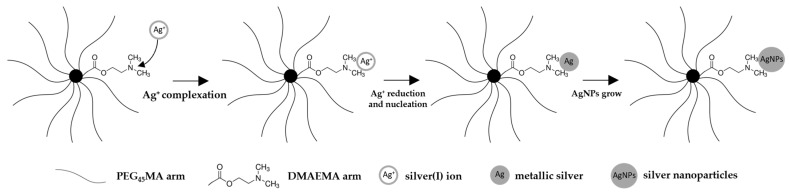
Scheme of the Ag nanoparticles formation process by reduction of silver ions on DMAEMA build-in of the structure of the star polymer.

The formation process of AgNPs on the functional star polymer was carried out in three different media (EtOH, EtOH:Ace, H_2_O:Ace) without an additional reducing agent and monitored by UV–Vis spectroscopy, which provides indirect but widely accepted evidence of nanoparticle formation [41,42,43]. The process was continued until the absorption of the characteristic band for AgNPs in the UV–Vis spectrum no longer increased. To avoid the influence of pH or AgNO_3_ concentration on AgNP formation, reactions were carried out in neutral solutions with a constant concentration of AgNO_3_. All three solutions changed color (from colorless through golden yellow to brown) during the reduction process. An absorption band at a wavelength of ~430 nm was observed, as can be seen in Figure 3a, which shows exemplary UV–Vis spectra obtained during the reduction process of silver ions for one of the tested solutions (H_2_O:Ace) of the STR_1. This absorption band corresponds to the surface plasmon resonance absorption peak of the nearly spherical or spherical silver nanoparticles, which indicates the formation of AgNPs [28,44]. This was achieved due to the presence of DMAEMA in the structure of star polymers. The free pair of electrons on the nitrogen atom in the tertiary amine group of the DMAEMA possesses coordination and reduction capabilities. Consequently, DMAEMA can act as both a complexing agent and a reducing agent. Initially, silver ions (Ag^+^) are trapped by DMAEMA through complexation with the nitrogen atoms, forming a (Ag^+^)-DMAEMA complex. Subsequently, the Ag^+^ ions are reduced in situ to form silver atoms during the nucleation stage. This nucleation process continues with the growth of silver crystals, ultimately resulting in the formation of silver nanoparticles (AgNPs) [28,29]. The Ag nanoparticle-formation process is schematically shown in Figure 4. Moreover, it was also reported that a hydrophilic PEO block can slowly reduce Ag^+^ ions into AgNPs without additional reductant [45,46]. The research by Ma and Zang [45,46] demonstrates that the AgNPs formed are mostly deposited on the polymer surface, and only a small fraction is present as free nanoparticles. Additionally, steric stabilization is also achieved due to the bulky, branched architecture of the star polymers, which creates a physical barrier around the nanoparticles. This combination of coordination bonding and steric hindrance contributes to the effective stabilization of AgNPs in the polymeric network.

The process of AgNP formation is faster and allows for obtaining a higher concentration of nanoparticles in the case of star polymers with a higher content of DMAEMA (STR_2) in their structure, as shown in the graph of the dependence of the maximum absorbance band characteristic for Ag nanoparticles on time for the tested solutions (Figure 3b. The maximum absorbance values of the characteristic absorption band for AgNP are higher for the corresponding solutions (H_2_O-Ace, EtOH, EtOH-Ace) containing STR_2 as a reducing agent than STR_1. The biggest difference is the most visible in the case of the water/acetone (H_2_O-Ace) solutions of the STR_1 and STR_2, for which absorbance is equal to A = 0.76 and A = 3.74, respectively. However, the difference in the maximum absorbance of silver nanoparticles in subsequent solutions of polymer stars (EtOH, EtOH-Ace) is less visible, and it reaches the lowest value for the EtOH-Ace solution: A_STR_1_ = 2.45, A_STR_2_ = 2.96. This observation confirms studies from Huang et al. [28], which show higher absorbance intensity for micelles with higher content of DMAEMA.

**Figure 5:**
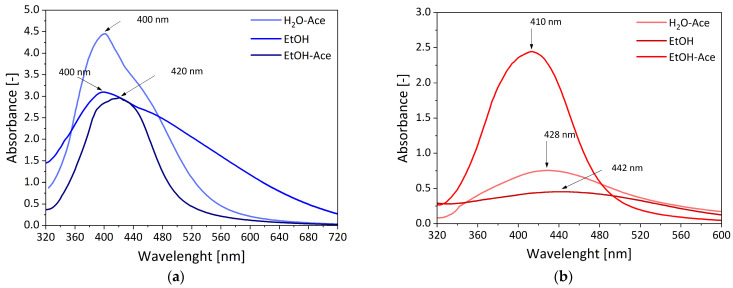
UV–Vis spectra obtained after the reduction of silver ions in the tested solutions containing star polymers: (**a**) STR_2; (**b**) STR_1. UV spectra were measured in quartz cuvette d = 0.1 cm at 25 °C.

The solution in which the silver salt-reduction process is carried out has a significant influence on the formation of AgNPs. It affects not only the duration of the process and its effectiveness but also the size of the obtained nanoparticles. In the EtOH medium, the AgNP generation was the slowest and lasted 10 days. It led to the formation of large nanoparticles (d_AgNPs_ ~70 nm), which indicates λ_max_ ~442 nm for STR_1, and λ_max_ ~400 nm, which indicates d_AgNPs_ ~35 nm for STR_2. A significantly faster reduction process occurred in H_2_O-Ace solution, and its duration was 5 days, which led to the formation of smaller AgNPs of d_AgNPs_ ~55 nm, with λ_max_ ~428 nm for STR_1 and d_AgNPs_ ~35 nm, λ_max_ ~400 nm for STR_2. Reactions carried out in EtOH-Ace solution lasted 7 days; nevertheless, the maximum wavelength shifted to lower values (λ_max_ ~410 nm) for STR_1 and higher values (λ_max_ ~420 nm) for STR_2, indicating the formation of smaller nanoparticles for STR_1 (d_AgNPs_ ~40 nm) and slightly larger nanoparticles for STR_2 (d_AgNPs_ ~45 nm). The size of particles was estimated based on wavelength according to previous studies [47,48]. The most effective process occurred in H_2_O-Ace solution for star polymers with a higher content of DMAEMA. Still, for STR_2, very efficient AgNP generation was observed in each solution (Figure 5a). For STR_1, the most efficient AgNP formation was noted in EtOH-Ace solution, and it was over three times more effective than in H_2_O-Ace solution and five times more effective than in ethanol (Figure 5b). The differences may be attributed to the subtle structural variations between the two star polymers. STR_2 has a higher number of arms and higher DMAEMA content compared to STR_1, which could result in a more uniform spatial distribution of functional groups capable of interacting with silver ions. This increased density and availability of tertiary amine groups in STR_2 may facilitate the more efficient and consistent complexation and reduction of Ag^+^ ions, regardless of the solvent environment.

In contrast, STR_1 may be more sensitive to solvent polarity and composition due to its lower functionality and potentially more heterogeneous distribution of reactive sites, which can influence the accessibility of Ag^+^ ions and the local reduction environment.

**Figure 6:**
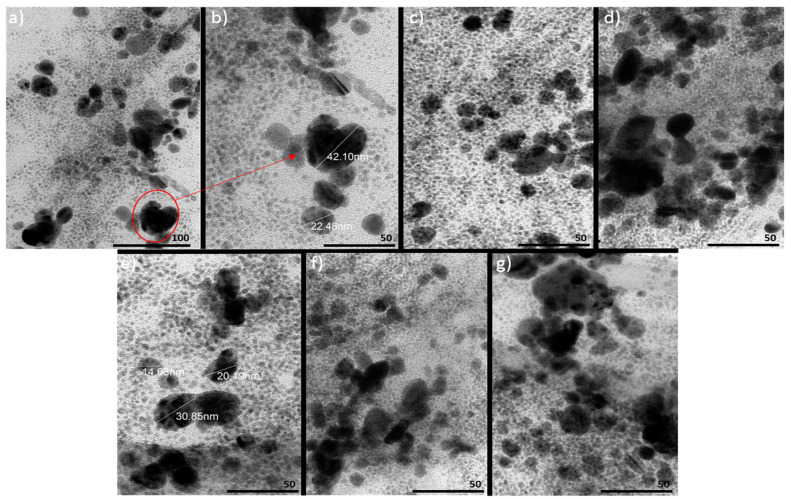
TEM images of AgNPs reduced on STR_2 in: H_2_O-Ace medium (**a**,**b**), EtOH-Ace medium (**c**) and EtOH (**d**) as well as STR_1 in: H_2_O-Ace medium (**e**), EtOH-Ace medium (**f**) and EtOH (**g**).

The silver salt-reduction process conducted in EtOH solution with STR_2 is characterized by the largest particle-size distribution (PSD), which was observed as a broad absorption band consisting of two maxima, with maximum absorbance for one peak λ_max1_ at ~400 nm and for the other peak λ_max2_ at ~440 nm. This suggests the generation of two aggregates of AgNPs with particles sizes of d_AgNPs_ ~35 nm and d_AgNPs_ ~70 nm, respectively. For the reduction on STR_2 in H_2_O-Ace medium, we observed a slight broadening of the peak around λ_max_ ~420, which could suggest the presence of two types of aggregates of AgNPs; this was confirmed by TEM analysis (Figure 6).

### 3.3. Transmission Electron Microscopy Analysis of the Morphology and Size Distribution of AgNPs

**Figure 7:**
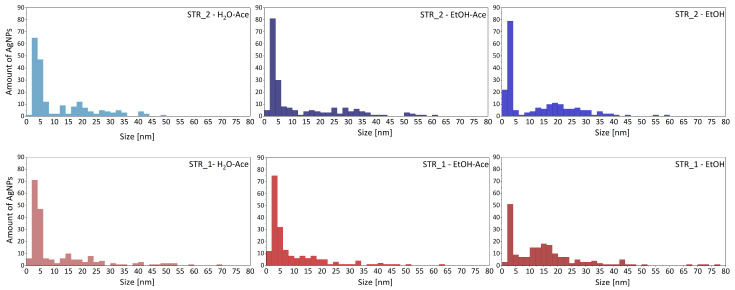
Particle size distribution calculated from TEM images of AgNPs dispersed in different solutions of STR_1 and STR_2: H_2_O-Ace, EtOH-Ace and EtOH.

TEM studies show different types of aggregates of AgNPs generated on STR_2 in H_2_O-Ace medium (Figure 6). Particles vary from very small (d_AgNPs_ ~2 nm) to larger aggregates—for instance, d_AgNPs_ ~22.5 nm and d_AgNPs_ ~40 nm. As can be seen from the graphs shown in Figure 7, which show the size distribution of silver nanoparticles in solutions of polymer stars STR_1 and STR_2, in all solutions, the presence of both particles with sizes below 10 nm and in the range of 10–50 nm is observed. Additionally, in the case of the ethanol solution of the polymer star STR_1, Ag nanoparticles 65–75 nm in size were detected. However, silver particles with sizes below 10 nm predominate, which indicates a low tendency of nanoparticles to form aggregates, due to the presence of the polymer star in the solution, which not only reduces silver ions but also maintains silver nanoparticles in its structure, preventing the fusion of nanoparticles. It is also evident that the solvent type affects aggregate formation, which is most pronounced in ethanol (EtOH), especially for STR_1. On the other hand, in mixtures of acetone with water or ethanol (H_2_O-Ace, EtOH-Ace), the formation of silver nanoparticle aggregates occurs to a lesser extent. The obtained results are analogous to those obtained by UV–Vis spectroscopy. Moreover, AgNPs exhibited spherical or spherical-like shapes, which also confirms the conclusions drawn from the studies conducted by UV–Vis spectroscopy.

### 3.4. Characterization of Bone Cements Modified with STR-AgNPs

Silver nanoparticles are widely studied as antibacterial agents in bone cements [35,36,37,38]. As Wekwejt et al. in their studies [37] showed, the addition of silver nanoparticles imparts antibacterial efficacy; however, it worsens cytotoxicity. These studies indicate great potential in the antibacterial activity of AgNPs in bone cements; however, free AgNPs may be toxic for cells. This led us to try to incorporate STR-AgNPs into acrylate bone cement. Both STR_1-AgNPs and STR_2-AgNPs were introduced to the bone cement (BC_STR_1-AgNPs and BC_STR_2-AgNPs). This modification improved compressive strength (σ_M (BC_STR_1-AgNPs)_ = 101.7 ± 1.3 MPa, σ_M (BC_STR_2-AgNPs)_ = 98.6 ± 4.4 MPa), while for un-modified bone cement, compressive strength was σ_M (BC)_ = 93.1 ± 5.1 MPa. Thus, the modification may impart antibacterial properties to bone cement without compromising mechanical strength. In this publication, we only show the effect of the modification on the mechanical properties of bone cements, but the promising results of this research will result in an extension of the scope of polymer stars introduced into cement and a broader characterization of their properties.

### 3.5. Evaluation of Antibacterial Activity of Prepared Materials

**Figure 8:**
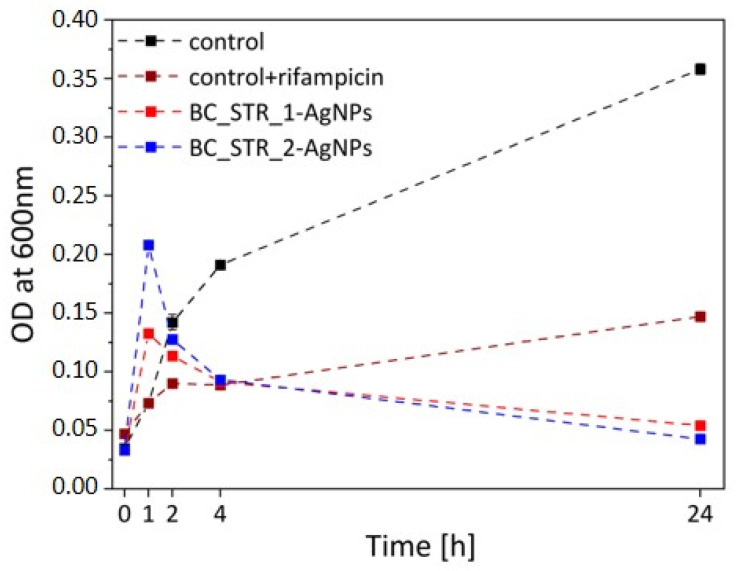
Growth curves of bacterial outgrowth after incubation with *E. coli*. Each point on the growth curve represents the average OD measured at 600 nm.

The determination of bacterial growth via optical density (OD) measurements is a well-established and widely used method in microbiological research. In our study, we followed the same protocol that has already been successfully applied and validated in our previous works [49,50]. To study the growth curve in the presence of BC_STR_1-AgNPs and BC_STR_2-AgNPs, optical density measurements at 600 nm (OD_600 nm_) of the bacterial culture were carried out. The graph presented below (Figure 8) compares the OD measurement (in triplicates) of bacterial cultures. The results provide insights into the antibacterial efficacy of these materials over a 24 h period.

All tested samples exhibited superior antibacterial activity against *E. coli* compared to the control suspension, both with and without the antibiotic Rifampicin. During the initial hour of observation, a transient increase in optical density was detected for BC_STR_1-AgNPs and BC_STR_2-AgNPs, suggesting initial bacterial adaptation or delayed nanoparticle interaction. However, following this phase, a consistent decline in OD was recorded, indicating a progressive reduction in bacterial viability. This bactericidal effect persisted over the 24 h monitoring period, confirming the sustained antimicrobial properties of the STR-AgNP-modified bone cements. Keshari et al. [51] also showed the strong antibacterial activity of AgNPs against *E. coli*. Moreover, Chen et al. [52] demonstrated the inhibition of bacterial growth when exposed to polymer composites with AgNPs.

## 4. Conclusions

Star polymers were successfully synthesized via ATRP using the “arm-first” method with molecular weights in the range *M*_n_ 15,000–16,000 and a relatively narrow dispersity of ~1.5. The introduction of a functional monomer (DMAEMA) into the star polymers enabled the efficient formation of AgNPs on STR in various solvents without additional reducing agents. The formation process, influenced by the solvent and DMAEMA content, was the fastest and most efficient in H_2_O-Ace, producing smaller nanoparticles. TEM confirmed the spherical shape of AgNPs ranging from ~2 nm to 40 nm. DMAEMA facilitated the complexation and in situ reduction of Ag^+^ ions, enhancing AgNP formation and stability. Incorporating STR-AgNPs into acrylate bone cement improved compressive strength (99 and 102 MPa compared to 93 MPa for non-modified cement), indicating potential antibacterial benefits without affecting mechanical properties. Moreover, antibacterial studies show strong antibacterial activity against *E. coli*. This study highlights the successful synthesis of functional star polymers and their application in generating stable AgNPs, with promising biomedical applications. Future research will further explore and characterize these materials.

## Figures and Tables

**Table 1:** Star polymer characteristics. ^a^ Conversion of monomer DMAEMA determined by GC. ^b^ Relative number-average molecular weights (*M*_n,app_) and dispersity (*Ð*) were determined by GPC analysis (THF as eluent) calibrated to PMMA standards. ^c^ Absolute number-average molecular weights (*M*_n,abs_) were determined by GPC (THF as eluent) equipped with a multi-angle laser light-scattering detector. ^d^ Number of arms was calculated as shown in Supplementary Materials.

Name	Conversion [%] ^a^	*M*_n,app_ ^b^	*M*_n,abs_ ^c^	*Đ* ^b^	No. of Arms ^d^
STR_1	86	16,100	23,900	1.42	47
STR_2	90	15,200	28,400	1.57	56

## Data Availability

The original contributions presented in this study are included in the article and Supplementary Materials. Further inquiries can be directed to the corresponding author.

## Supplementary Materials

The following supporting information can be downloaded at: https://www.mdpi.com/article/10.3390/ma18133009/s1, Figure S1: ^1^H NMR spectrum of STR measured in D_2_O at 295K.
