# Supplementary material for: Star Polymers as a Reducing Agent of Silver Salt and a Carrier for Silver Nanoparticles
Source: Materials (Basel). 2025 Jun 25;18(13):3009. doi: 10.3390/ma18133009 (PMC12251048; doi:10.3390/ma18133009)
Supplement: Supplementary file 1 [file materials-18-03009-s001.zip › materials-3645397-supplementary.pdf]

Star polymers as a reducing agent of silver salt and a carrier for silver nanoparticles – supplementary material

Katarzyna Szcześniak[1]*, Grzegorz Przesławski[1], Jakub Kotecki[1], Weronika Andrzejewska[2], Katarzyna Fiedorowicz[2], Marta Woźniak-Budych[2], Maciej Jarzębski[3]*, Piotr Gajewski[1] and Agnieszka Marcinkowska[1]

Star polymer characterization – nuclear magnetic resonance (NMR)

1. Experimental

^{1}H NMR spectra were acquired with Bruker Avance III 600 spectrometer operating at 600.27 MHz. The width of ^{1}H NMR 90° pulse was 18 µs, relaxation delay 10 s, acquisition time 2.73 s. Spectra of polymer samples were measured with at least 64 scans. Samples were acquired at 295K using D_2O as a solvent.

2. Results

The ^{1}H NMR spectrum of the star polymer, along with its structural representation and corresponding signal assignments, recorded in D_2O at 295 K, is presented in Figure S1. The spectrum exhibits characteristic resonances corresponding to protons from both DMAEMA and EGDM repeating units. DMAEMA segments are observed at δ = 4.20 ppm (**5**), δ = 2.80 ppm (**6**), and δ = 2.35 ppm (**7**). The PEGM signals are observed at δ = 3.70 ppm (**3**) and δ = 3.39 ppm (**4**) and EGDM signals are observed at δ = 3.70 ppm (**8**). Signals in the range δ = 0.40–1.70 ppm (**2**) corresponds to the methyl groups of the polymer backbone, whereas the signal in the δ = 1.70–2.40 ppm region (**1**), is ascribed to backbone methylene protons.

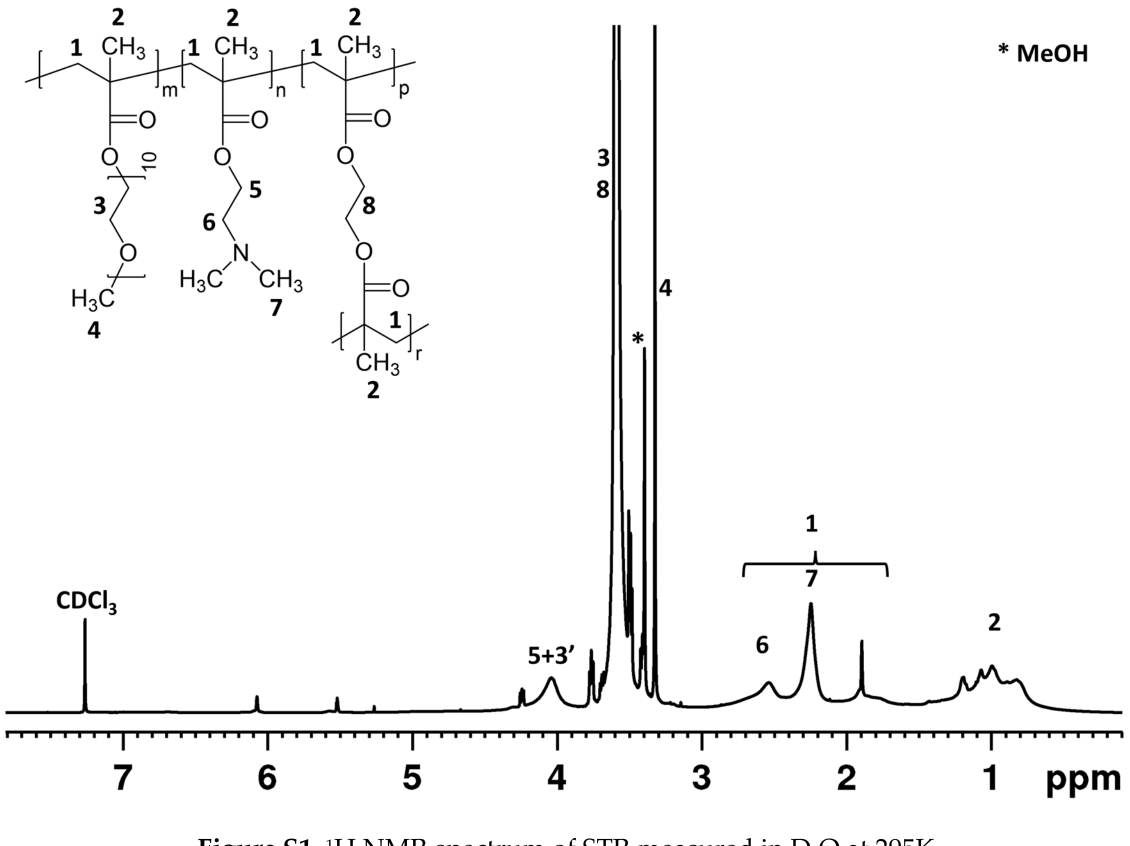

Figure S1. ^{1}H NMR spectrum of STR measured in D_2O at 295K.
